# Association of Pre-stroke Frailty With Prognosis of Elderly Patients With Acute Cerebral Infarction: A Cohort Study

**DOI:** 10.3389/fneur.2022.855532

**Published:** 2022-05-30

**Authors:** Fuxia Yang, Nan Li, Lu Yang, Jie Chang, Aijuan Yan, Wenshi Wei

**Affiliations:** Department of Neurology, Huadong Hospital Affiliated to Fudan University, Shanghai, China

**Keywords:** frailty, frailty index, stroke, cerebral infarction, mRS, NIHSS

## Abstract

**Background:**

Frailty is a state of cumulative degradation of physiological functions that leads to adverse outcomes such as disability or mortality. Currently, there is still little understanding of the prognosis of pre-stroke frailty status with acute cerebral infarction in the elderly.

**Objective:**

We investigated the association between pre-stroke frailty status, 28-day and 1-year survival outcomes, and functional recovery after acute cerebral infarction.

**Methods:**

Clinical data were collected from 314 patients with acute cerebral infarction aged 65–99 years. A total of 261 patients completed follow-up in the survival cohort analysis and 215 patients in the functional recovery cohort analysis. Pre-stroke frailty status was assessed using the FRAIL score, the prognosis was assessed using the modified Rankin Scale (mRS), and disease severity using the National Institutes of Health Stroke Scale (NIHSS).

**Results:**

Frailty was independently associated with 28-day mortality in the survival analysis cohort [hazard ratio (HR) = 4.30, 95% *CI* 1.35–13.67, *p* = 0.014]. However, frailty had no independent effect on 1-year mortality (*HR* = 1.47, 95% *CI* 0.78–2.79, *p* = 0.237), but it was independently associated with advanced age, the severity of cerebral infarction, and combined infection during hospitalization. Logistic regression analysis after adjusting for potential confounders in the functional recovery cohort revealed frailty, and the NIHSS score was significantly associated with post-stroke severe disability (mRS > 2) at 28 days [pre-frailty adjusted odds ratio (a*OR*): 8.86, 95% *CI* 3.07–25.58, *p* < 0.001; frailty a*OR*: 7.68, 95% *CI* 2.03–29.12, *p* = 0.002] or 1 year (pre-frailty a*OR*: 8.86, 95% *CI* 3.07–25.58, *p* < 0.001; frailty a*OR*: 7.68, 95% *CI* 2.03–29.12, *p* = 0.003).

**Conclusions:**

Pre-stroke frailty is an independent risk factor for 28-day mortality and 28-day or 1-year severe disability. Age, the NIHSS score, and co-infection are likewise independent risk factors for 1-year mortality.

## Introduction

Stroke has become the second largest cause of death and the third largest cause of disability after ischemic heart disease and is an important factor in disability-adjusted life-years (DALYs) lost in people over 50 years old (1). In China, the prevalence of stroke exceeds that of ischemic heart disease, with more than 2 million new cases per year, making stroke the most DALYs lost among all diseases (2). Although measures such as endovascular intervention and the establishment of stroke centers have significantly reduced the mortality of the cerebrovascular disease, surviving patients have also increased the social disability burden (3). Functional recovery tended to stabilize at 3–6 months after stroke, but the recovery of different patients still showed individual differences, and some patients had accelerated accumulation of disabilities over time (4–6).

Frailty status is a meaningful manifestation of aging in the population, characterized by a decline in function across multiple physiological systems. This decline is a disproportionate change in health status caused by small stress events accompanied by an increased vulnerability to stressors (7). Frailty is more prone to negative outcomes and is a predictor of all-cause mortality (8, 9). Acute cerebral infarction produces a major impact on the body and makes patients more prone to adverse events, such as poststroke pneumonia (10), persistent disability (11), and neurocognitive disorders (12).

Shanghai, the country's most populous city, has 3,824,400 registered residents aged 65 years and above in 2020, nearly 25.9% of the population (13). There are 40 large-scale general hospitals in Shanghai. A cross-sectional study based on Fried's frailty phenotype was used to assess frailty status was performed in 780 Shanghai suburban older adults aged 65–74 years in 2019. The percentages of robust, pre-frail, and frail were 48.46, 47.69, and 3.85% (14).

This study aimed to establish the relationship between pre-stroke frailty and outcomes after acute ischemic stroke. We divided participants into survival and functional recovery cohorts and explored 28-day and 1-year post-stroke outcomes.

## Methods

### Study Design and Participants

This cohort study enrolled 314 consecutive older adult patients with acute cerebral infarction at Huadong Hospital affiliated with Fudan University from September 2019 to September 2020. The inclusion criterion was patients between 65 and 99 years. All patients underwent CT/MRI after the symptoms occurred, and lesions of acute ischemic stroke were found on CT/MRI. Exclusion criteria included (1) stroke symptom onset for more than 1 week, (2) only clinical symptoms without imaging evidence, (3) functional impairment prior to the onset of stroke [modified Rankin Scale (mRS) ≥ 3], and (4) living alone and unable to complete the questionnaire independently. Furthermore, 32 patients who had consciousness disorders (*n* = 15), severe cognitive dysfunction (*n* = 11), and aphasia (*n* = 6) would not provide the medical history, and the relevant information would be provided by the guardian or caregiver. If the caregiver was unable to remember illnesses completely, the researchers would get supplementary medical history through the test report.

Participants or guardians were fully informed of the purpose and content of the study in written and oral form, and this study was approved by the Ethics Committee of the Huadong Hospital affiliated with Fudan University.

### Clinical and Demographic Data

Patient clinical characteristics, such as age, sex, body mass index (BMI), lesion side of stroke, Trial of Org 10172 in Acute Stroke Treatment (TOAST) classification, National Institutes of Health Stroke Scale (NIHSS), comorbidities (previous stroke, hypertension, diabetes, dyslipidemia, atrial fibrillation, and smoking), stroke treatment (antiplatelet therapy, intravenous thrombolysis, or thrombolysis), and concurrent infection during hospitalization (co-infection).

### Prognostic Assessment

The WHO recommends the mRS (15) to assess post-stroke prognosis, defined as follows: (1) mRS = 0 (no symptoms at all); (2) mRS = 1 (no significant disability): despite symptoms, able to carry out all usual duties and activities; (3) mRS = 2 (slight disability): unable to perform all previous activities but able to look after own affairs without assistance; (4) mRS = 3 (moderate disability): requiring some help but able to walk without assistance; (5) mRS = 4 (moderately severe disability): unable to walk without assistance and unable to attend to own bodily needs without assistance; (6) mRS = 5 (severe disability): bedridden, incontinent, and requiring round-the-clock nursing care and attention; (7) mRS = 6 (death) (16). An mRS score of ≤ 2 was considered to have a favorable prognosis (no symptoms or slight disability), while mRS > 2 was considered to have a worse prognosis (severe disability). An mRS score of 6 is equivalent to death. Survival outcome and functional recovery status were assessed using mRS at 28 days and 1 year after disease onset.

### Assessment of Pre-stroke Frailty Status

The two main frailty assessment instruments are the frailty phenotype and the frailty index (deficit accumulation) (17). Since routine measurement tools require tests such as grip strength or walking speed, these are difficult to evaluate in patients with limb hemiplegia due to cerebral infarction. Instead, this study used the FRAIL scale to assess pre-stroke frailty status, a scale based on self-report that does not require activities affected by the disease itself (18). Existing studies confirm that the FRAIL scale has high sensitivity and specificity compared with the phenotype criteria (18), which is the same scale usually applied in the Chinese population (19, 20). The FRAIL scale consists of five components: fatigue, resistance, ambulation, illness, and loss of weight. It involves asking about the frequency of fatigue occurrence during the past 4 weeks, resistance by asking whether one can climb a staircase independently, ambulation by asking whether one can walk for 100 m independently, illness by asking whether one has more than five or more diseases, and weight loss by asking whether one lost 5% of their weight or more within 6 months. We collected details of fatigue from caregivers whether the patient complained of six somatic symptoms more than half the time in the past 4 weeks. They included the presence of muscle pain or tired muscles after activity, the need to sleep longer, poor sleep, prolonged tiredness after activity, and poor concentration (21). The FRAIL scale scores range from to 0–5 (0 = best to 5 = worst) and represent frailty (3–5), pre-frailty (1, 2), and robust (0) health status (22).

### Follow-Up and Outcome Events

Follow-up visits were conducted face-to-face or *via* telephone, with telephone follow-up being done by professionally trained personnel. Follow-up was performed at 28 days and 1 year after the stroke. Then, 1-year follow-up was allowed with an error of not more than 7 days. All patients received face-to-face follow-up at 28 days. In addition, 61 patients or their respective caregivers received telephone follow-up and others visited the clinic at 1 year. The characteristics of patients who did not arrive at the clinic for follow-up were as follows: died (*n* = 31), bedridden (*n* = 22), and others (*n* = 8). All patients who completed the follow-up visit were included in the survival cohort analysis, and those who survived at 1 year were included in the functional recovery cohort analysis.

### Statistics

We divided our study population into two non-interfering groups according to survival status at 1 year. Between-group differences in baseline were obtained using the Kruskal–Wallis test or the Pearson *X*^2^ test. All variables significantly associated with outcome in the univariate analysis were included in a multivariable binary Cox or logistic regression analysis, and the hazard ratio (*HR*) or the adjusted odds ratios (a*OR*) and 95% confidence intervals (95% *CI*s), where the 1-year functional improvement rate was determined, were calculated using the Pearson *X*^2^ test and pair comparisons were performed. The level of significance was set at *p* < 0.05. Statistical analyses were performed using SPSS version 25.0.

## Results

### Study Process and Grouping Based on Frailty Status

**Figure 1:**
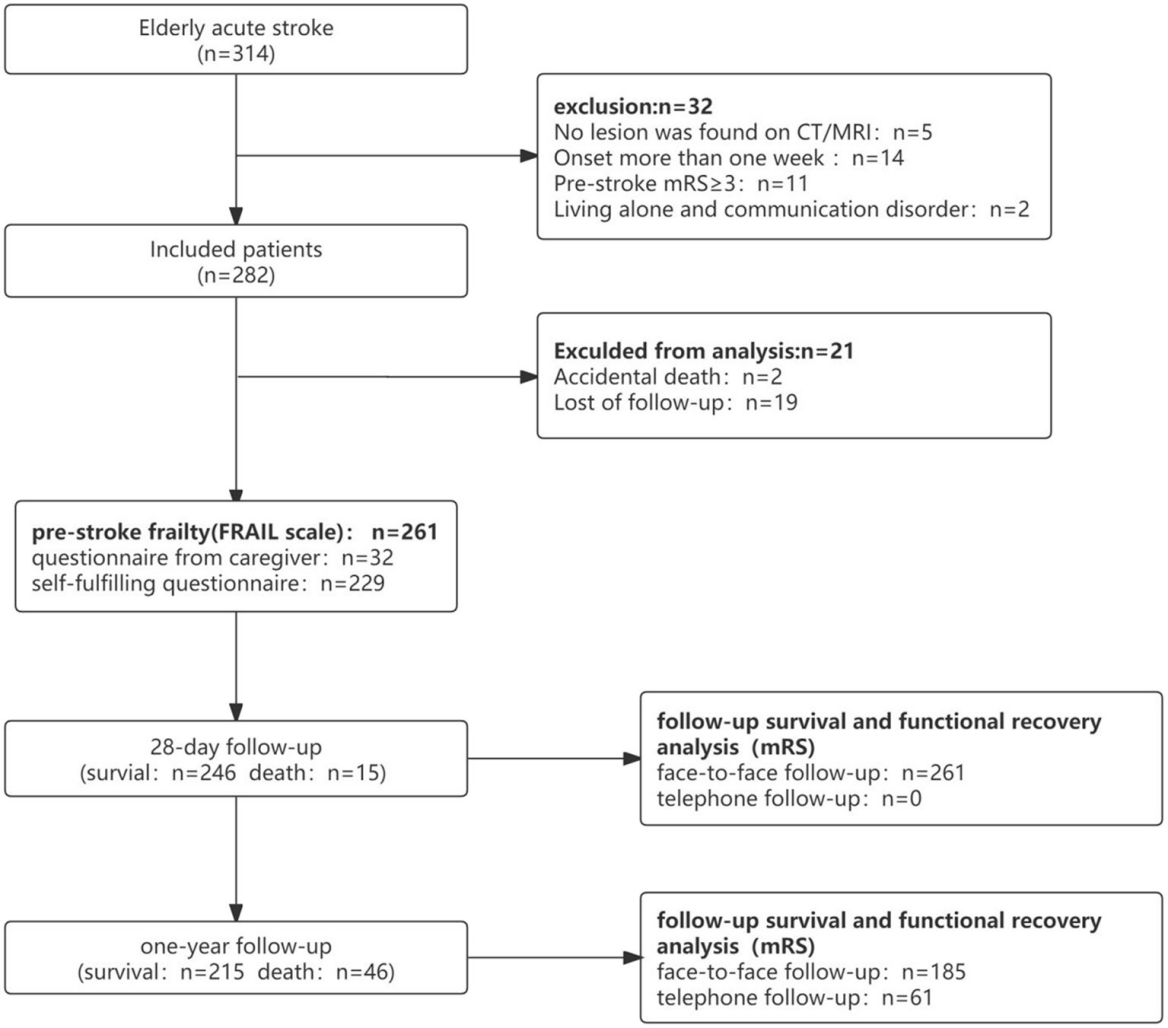
Diagram of the study design.

**Figure 2:**
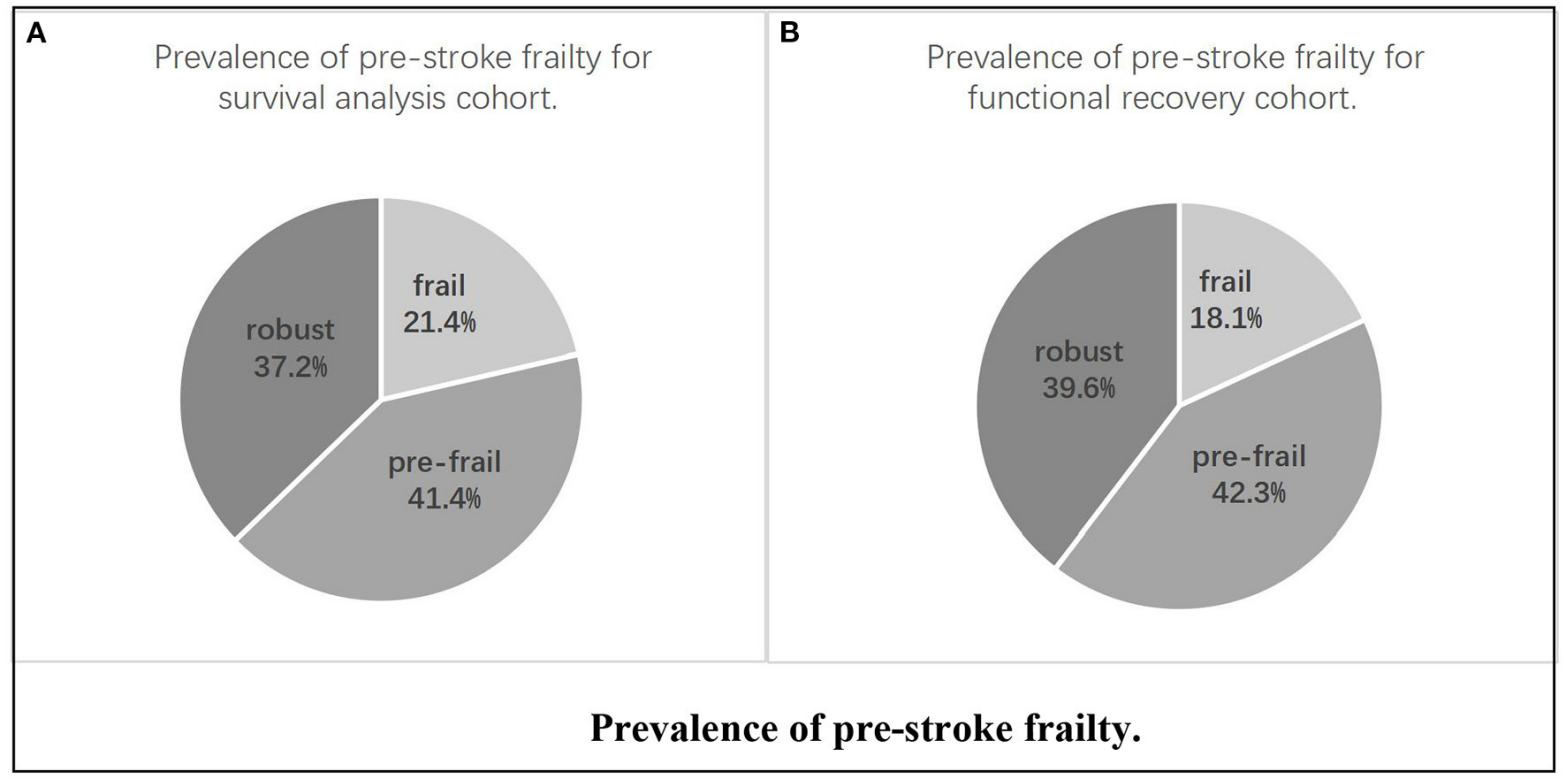
Prevalence of pre-stroke frailty. **(A)** Prevalence of pre-stroke frailty for survival analysis cohort. **(B)** Prevalence of pre-stroke frailty for functional recovery cohort.

Clinical data of 314 patients with acute cerebral infarction aged 65–99 years were included. In total, 261 completed follow-up visits were entered into the survival cohort analysis, and 215 were included in the functional recovery cohort analysis (Figure 1). In the survival cohort, the prevalence of frailty status before stroke was as follows: 97 (37.2%) were robust, 108 (41.4%) were pre-frail, and 56 (21.4%) were frail. Due to sample size limitations, we combined robust and pre-frail status as non-frail. The 261 patients were further divided into two groups according to the frailty dichotomy: 205 (78.6%) were non-frail and 56 (21.4%) were frail. In the functional recovery cohort, the prevalence of frailty status was as follows: 85 (39.6%) were robust, 91 (42.3%) were pre-frail, and 29(18.1%) were frail (Figure 2).

### Demographic Data in the Survival Analysis Cohort

Demographic data from the survival analysis cohort were recorded. The frail group had significantly higher age (*p* < 0.001), NIHSS scores (*p* = 0.032), and possibility of co-infection (*p* = 0.005) than the non-frail group (Table 1). There were 15 patients who died within 28 days, with 6 patients (2.9%) in the non-frail group and nine patients (16.1%) in the frail group (*X*^2^-value = 14.030, *p* < 0.001), while 46 patients died within 1 year, 29 (14.1%) in the non-frail group and 17 (30.4%) in the frail group (*X*^2^-value = 7.962, *p* = 0.005) (Table 2).

**Table 1:** Demographics and clinical characteristics of the participants for survival analysis.

	**Non-frail** **(*n* = 205)**	**Frail** **(*n* = 56)**	***P*-value**
Age [year, median (IQR)]	74(70–80)	78(72–86)	<0.001^*^
BMI [kg/m^2^,median (IQR)]	24.6(22.2–26.2)	24.4(22.3–26.6)	0.686
NIHSS score [median (IQR)]	3(1–5)	3(1–7)	0.032
Sex			0.130
Male (%)	122(46.7)	27(10.4)	
Female (%)	83(31.8)	29(11.1)	
TOAST classification			0.004^*^
Atherosclerotic (%)	48(18.4)	15(5.7)	
Lacunar (%)	72(27.6)	10(3.8)	
Cardioembolic (%)	40(15.3)	18(7.0)	
Unknown (%)	43(16.5)	9(3.4)	
Other (%)	2(0.8)	4(1.5)	
Side of lesion			0.587
Left (%)	96(36.8)	27(10.3)	
Right (%)	77(29.9)	18(6.9)	
Both (%)	30(11.5)	11(4.2)	
Stroke treatment			0.835
Antiplatelettherapy (%)	185(70.9)	52(19.9)	
Intravenous thrombolysis (%)	15(5.8)	3(1.1)	
Thrombectomy (%)	5(1.9)	1(0.4)	
**Previous stroke (%)**			
Smoking	58(22.2)	20(7.6)	0.282
Former (%)			0.047^*^
Current (%)	33(12.6)	2(0.8)	
Never (%)	25(9.6)	9(3.4)	
Hypertension (%)	147(56.4)	45(17.2)	
Diabetes mellitus (%)	161(61.7)	50(19.2)	0.070
Hyperlipidemia (%)	87(33.3)	25(9.6)	0.175
Atrial fibrillation (%)	54(20.7)	15(5.7)	0.947
Concurrent infection (%)	45(17.2)	19(7.3)	0.065
	43(16.5)	22(8.4)	0.005^*^

* *Indicates a signicant difference (p < 0.05) between non-frail and frail*.

**Table 2:** Survival status for 28-day and 1-year follow-up for survival analysis.

	**All**	**Non-frail**	**Frail**	***X*** ^**2**^ **-test**	
	**(*n* = 261)**	**(*n* = 205)**	**(*n* = 56)**	***X*** ^**2**^ **-value**	***p*-value**
28-day survival status					
Survival	246	199(97.1%)	47(83.9%)	14.030	<0.001^*^
Death	15	6(2.9%)	9(16.1%)		
One-year survival status					
Survival	215	176(85.9%)	39(69.6%)	7.962	0.005^*^
Death	46	29(14.1%)	17(30.4%)		

*NIHSS, National Institutes of Health Stroke Scale*.

*Values are shown as median (interquartile range) or ordinal variables and counts (%) for categorical variables*.

*Non-frail includes robust and pre-frail*.

* *Indicates a significant difference (p < 0.05) between non-frail and frail*.

### Risk Factors for Mortality in the Survival Analysis Cohort

Factors with intergroup differences *p* < 0.1 in the survival analysis cohort were included in the Cox regression, such as age, co-infection, frailty status, hypertension, atrial fibrillation, NIHSS score, TOAST classification, smoking history, and sex (23). In the 28-day survival analysis, frailty status (*HR* = 4.30, 95% *CI* 1.35–13.67, *p* = 0.014) and NIHSS score (*HR* = 1.09, 95% *CI* 1.02–1.18, *p* = 0.019) were independently associated with mortality. Frailty had no independent association with mortality within 1 year (*HR* = 1.47, 95% *CI* 0.78–2.79, *p* = 0.237), and those independently associated with mortality within 1 year were age (*HR* = 1.07, 95% *CI* 1.03–1.12, *p* = 0.001), NIHSS score (*HR* = 1.07, 95% *CI* 1.02–1.12, *p* = 0.006), and co-infection (*HR* = 0.18, 95% *CI* 0.08–0.41, *p* < 0.001) (Table 3).

**Table 3:** Cox regression for 28-day and 1-year follow-up survival status for survival analysis.

	**28-day survival status**		**One-year survival status**	
	**HR(95%CI)**	***P*-value**	**HR(95%CI)**	***P*-value**
Age	1.09(0.95–1.10)	0.616	1.07(1.03–1.12)	0.001^*^
Sex	0.73(0.24–2.25)	0.582	0.73(0.37–1.46)	0.374
Concurrent infection	0.32(0.06–1.67)	0.178	0.18(0.08–0.41)	<0.001^*^
NIHSS	1.09(1.02–1.18)	0.019^*^	1.07(1.02–1.12)	0.006^*^
Frailty status	4.30(1.35–13.67)	0.014^*^	1.47(0.78–2.79)	0.237
Atrial fibrillation	1.46(0.26–8.26)	0.672	0.95(0.29–3.07)	0.931
Hypertension	1.15(0.28–4.67)	0.845	1.35(0.65–2.79)	0.420
Smoking				
Former	Reference	-	Reference	-
Smoking current	0.82(0.80–8.37)	0.867	0.86(0.24–3.06)	0.819
Smoking never	0.00(0.00–2.43)	0.935	0.50(0.11–2.20)	0.356
TOAST classification				
Atherosclerotic	Reference	-	Reference	-
Lacunar	0.00(0.00–5.99)	0.967	0.21(0.03–1.77)	0.152
Cardioembolic	2.04(0.32–13.22)	0.453	1.01(0.29–3.46)	0.991
Unknown	4.25(0.60–30.00)	0.147	2.30(0.85–6.28)	0.102
Other	1.63(0.13–20.47)	0.706	0.90(1.88–4.36)	0.899

* *Indicates a significant difference (p < 0.05)between the groups*.

### Demographic Data of the Functional Recovery Cohort

Demographic data from the functional recovery cohort were recorded (Table 4). The prognosis of acute cerebral infarction was assessed by mRS (scored from 0 to 5). NIHSS score and frailty status were significantly different between groups (robust, pre-frail, and frail) at 28 days (*X*^2^ = 38.180, *p* < 0.001) or 1 year (*X*^2^ = 56.091, *p* < 0.001) (Table 5).

**Table 4:** Demographics and clinical characteristics of the participants for functional recovery analysis.

	**Robust** **(*n* = 85)**	**Pre-frail** **(*n* = 91)**	**Frail** **(*n* = 39)**	***P*-value**
Age [year, median (IQR)]	75(70–79)	74(69–80)	78(73–86)	0.005^*^
BMI [kg/m^2^, median (IQR)]	24.8 (23.3–26.1)	24.4 (22.1–25.8)	24.6 (22.8–26.1)	0.181
NIHSS score[median (IQR)]	2(1–6)	4(2–7)	4(2–10)	0.005^*^
Sex				0.297
Male (%)	54(25.1)	53(24.7)	19(8.8)	
Female (%)	31(14.4)	38(17.7)	20(9.3)	
TOAST classification				0.022^*^
Atherosclerotic (%)	15(7.0)	23(10.7)	9(4.2)	
Lacunar (%)	32(14.9)	39(18.1)	10(4.7)	
Cardioembolic (%)	14(6.5)	12(5.6)	13(6.0)	
Unknown (%)	24(11.2)	15(7.0)	5(5.2)	
Other (%)	0(0.0)	2(0.9)	2(0.9)	
Side of lesion				0.148
Left (%)	49(22.8)	36(16.7)	19(8.8)	
Right (%)	30(14.0)	41(19.1)	16(7.4)	
Both (%)	6(2.8)	14(6.5)	4(4.2)	
Stroke treatment				0.092
Antiplatelet therapy (%)	77(35.8)	82(38.1)	35(16.3)	
Intravenous Thrombolysis (%)	8(3.7)	5(2.3)	4(1.9)	
Thrombectomy (%)	0(0.0)	4(1.9)	0(0.0)	
Previous stroke (%)
Smoking	18(8.4)	33(15.3)	15(7.0)	0.049^*^
Former (%)	5(2.3)	3(1.4)	3(1.4)	0.248
Current (%)	18(8.4)	27(12.6)	5(2.3)	
Never (%)	62(28.8)	61(28.4)	31(14.4)	
Hypertension (%)	69(32.1)	71(35.8)	36(16.7)	0.150
Diabetes mellitus (%)	31(14.4)	44(20.5)	2(9.3)	0.175
Hyperlipidemia (%)	25(11.6)	23(10.7)	12(5.6)	0.753
Atrial fibrillation (%)	18(8.4)	13(6.0)	15(6.9)	0.009^*^
Concurrent infection (%)	4(1.9)	17(7.9)	10(4.6)	0.003^*^

* *Indicates a signicant difference (p < 0.05) between non-frail and frail*.

**Table 5:** The modified Rankin Scale (mRS) for each group of frailty status for a 28-day functional outcome and a 1-year follow-up functional outcome for functional recovery analysis.

	**All subjects** **(*n* = 215)**	**Robust** **(*n* = 85)**	**Pre-frail** **(*n* = 91)**	**Frail** **(*n* = 39)**	***X*** ^**2**^	***P*-value**
28-day functional outcome (%)					38.180	<0.001^*^
mRS = 0	18	14(6.5)	4(1.9)	0(0.0)		
mRS = 1	32	16(7.4)	10(4.7)	6(2.8)		
mRS = 2	52	30(14.0)	17(7.9)	5(2.3)		
mRS = 3	44	11(5.1)	23(10.7)	10(4.7)		
mRS = 4	36	9(4.2)	20(9.3)	7(3.3)		
mRS = 5	33	5(2.3)	17(7.9)	11(5.1)		
One-year functional outcome(%)					56.091	<0.001^*^
mRS = 0	57	40(18.6)	13(6.0)	4(1.9)		
mRS = 1	48	25(11.6)	20(9.3)	3(1.4)		
mRS = 2	32	7(3.3)	17(7.9)	8(3.7)		
mRS = 3	31	6(2.8)	17(7.9)	8(3.7)		
mRS = 4	22	5(2.3)	12(5.6)	5(2.3)		
mRS = 5	25	2(0.9)	12(5.6)	11(5.1)		

*NIHSS, National Institutes of Health Stroke Scale; mRS, modified Rankin Scale*.

*Values are shown as median (interquartile range) or ordinal variables and counts (%) for categorical variables*.

* *Indicates a significant difference (p < 0.05) between the groups*.

### Influencing Factors of Functional Recovery Analysis

Factors analyzed at *p* < 0.1 in the functional recovery cohort included multivariate logistic regression analysis for 28-day and 1-year functional recovery. These include age, NIHSS score, previous history of stroke, atrial fibrillation, co-infection, frailty status, TOAST classification, stroke treatment, and sex (23). An mRS score ≤ 2 was considered to have a favorable prognosis (no symptoms or slight disability), and an mRS score >2 was considered to have a worse prognosis (severe disability).

After adjusting for confounding variables (age, sex, and stroke risk factors), the presence of pre-stroke frailty and NIHSS score remained an independent predictor of negative prognosis at 28 days and 1 year. In the 28-day functional recovery analysis, the NIHSS score had an a*OR* of 2.06 (95% *CI* 1.63–2.60, *p* < 0.001), pre-frailty a*OR* of 8.86 (95% *CI* 3.07–25.58, *p* < 0.001), and frailty a*OR* of 7.68 (95% *CI* 2.03–29.12, *p* = 0.003) (Table 6), while in the 1-year analysis, the NIHSS score a*OR* of 1.43 (95% *CI* 1.24–1.63, *p* < 0.001), pre-frailty a*OR* of 5.14 (95% *CI* 2.00–13.20, *p* = 0.001), and frailty a*OR* of 9.28 (95% *CI* 2.85–30.18, *p* < 0.001) (Table 7).

**Table 6:** Logistic regression for a 28-day functional outcome for functional recovery analysis.

	**Unadjusted OR(95%CI)**	***P*-value**	**Adjusted OR(95%CI)**	***P*-value**
Age	1.02(0.97–1.06)	0.230	1.02(0.95–1.08)	0.595
Sex	1.39(0.80–2.39)	0.242	1.72(0.70–4.22)	0.240
Concurrent infection	0.06(0.01–0.25)	<0.001^*^	0.81(0.08–8.28)	0.861
Stroke history	1.60(0.89–2.89)	0.117	1.34(0.55–3.27)	0.512
Atrial fibrillation	0.40(0.21–0.81)	0.011	0.17(0.02–1.63)	0.125
NIHSS	1.90(1.58–2.29)	<0.001^*^	2.06(1.63–2.60)	<0.001^*^
**Frailty status**				
Robust	Reference	–	Reference	–
Pre-frail	4.65(2.46–8.78)	<0.001^*^	8.86(3.07–25.58)	<0.001^*^
Frail	6.11(2.64–14.14)	<0.001^*^	7.68(2.03–29.12)	0.003^*^
**TOAST classification**				
Atherosclerotic	Reference	–	Reference	–
Lacunar	0.03(0.12–0.53)	<0.001^*^	1.82(0.50–6.66)	0.368
Cardioembolic	1.20(0.47–3.02)	0.709	0.50(0.37–6.86)	0.607
Unknown	0.43(0.18–1.00)	0.051	3.44(0.78–15.09)	0.102
Other	–	0.999	–	0.999
**Stroke treatment**				
Antiplatelet therapy	Reference	–	Reference	–
Intravenous thrombolysis	1.63(0.57–4.66)	0.360	1.52(0.26–9.02)	0.654
Thrombectomy	–	0.999	–	0.999

* *Indicates a signicant difference (p < 0.05) between non-frail and frail*.

**Table 7:** Logistic regression for 1-year follow-up on functional outcomes for functional recovery analysis.

	**Unadjusted OR(95%CI)**	***P*-value**	**Adjusted OR(95%CI)**	***P*-value**
Age	1.04(1.00–1.08)	0.053	1.03(0.97–1.09)	0.311
Sex	1.15(0.66–2.02)	0.622	1.13(0.51–2.54)	0.757
Concurrent infection	0.08(0.03–0.21)	<0.001^*^	0.59(0.15–1.41)	0.464
stroke history	1.33(0.73–2.41)	0.348	1.02(0.46–2.28)	0.887
Atrial fibrillation	0.54(0.28–1.05)	0.068	2.40(0.23–25.13)	0.853
NIHSS	1.30(1.20–1.41)	<0.001^*^	1.43(1.24–1.63)	<0.001^*^
Frailty status				
Robust	Reference	-	Reference	-
Pre-frail	4.54(2.21–9.34)	<0.001^*^	5.14(2.00–13.20)	0.001^*^
Frail	8.86(3.70–21.25)	<0.001^*^	9.28(2.85–30.18)	<0.001^*^
Tostal system				
Atherosclerotic	Reference	-	Reference	-
Lacunar	0.24(0.11–0.52)	<0.001^*^	1.01(0.33–3.03)	0.991
Cardioembolic	1.12(0.48–2.62)	0.797	2.87(0.23–35.32)	0.411
Unknown	0.40(0.17–0.95)	0.039	1.25(0.38–4.13)	0.712
Other	–	0.999	–	0.999
Stroke treatment				
Antiplatelet therapy	Reference	-	Reference	-
Intravenous thrombolysis	0.58(0.18–1.86)	0.357	0.18(0.03–1.04)	0.057
Thrombectomy	2.60(0.42–15.92)	0.302	1.34(0.21–7.67)	0.069

* *Indicates a significant difference (p < 0.05) between the groups*.

### Functional Improvement Within 1 Year in the Functional Recovery Analysis Cohort

Of the 215 patients in the functional recovery cohort, 123 had a worse prognosis (mRS > 2), with 27 in the robust group, 64 in the pre-frail group, and 32 in the frail group at 28 days. If the functional status was assessed as mRS ≤ 2 at 1-year follow-up, it will be considered a significant functional improvement. As the degree of frailty increased, the likelihood of functional improvement after 1 year was low, with 13 (48.14%) considered robust, 21 (32.81%) considered pre-frail, and 5 (15.65%) considered frailty (*X*^2^ = 7.2, *p* = 0.27) after 1 year (Table 8).

**Table 8:** Functional improvement after 1 year follow-up for functional recovery analysis.

	**All**	**No improvement(%)**	**Improvement(%)**	***X*** ^**2**^ **-text**	
	**(*n* = 123)**	**(*n* = 84)**	**(*n* = 39)**	***X*** ^**2**^ **-value**	***P*-value**
Robust	27	14(51.86%)	13(48.14%)	7.2	0.027^*^
Pre-frail	64	43(67.19%)	21(32.81%)		
Frail	32	27(84.37%)	5(15.63%)		

* *Indicates a significant difference (p < 0.05) between the groups*.

## Discussion

This cohort study revealed the relationship between pre-stroke frailty status and acute cerebral infarction prognosis and found that pre-stroke frailty was an independent influencing factor for 28-day mortality but not for 1-year mortality. Besides, 1-year mortality was independently associated with advanced age, NIHSS score, and co-infection. Pre-stroke frailty status was associated with severe disability at 28 days and 1 year, suggesting possible negative functional improvement for those with frailty.

Previous studies have confirmed that pre-stroke frailty status is associated with adverse outcomes, such as stroke severity (24), mortality (25), short-term functional outcome (26), lower daily activity ability (27), discharge location (28), and post-stroke cognitive impairment (29). This study confirmed that pre-stroke frailty status exacerbated the risk of short-term mortality but had no independent effect on long-term mortality. Long-term mortality outcomes after stroke are largely caused by diseases other than stroke (30). Our study also confirmed that the main causes of mortality in patients with early death were cerebral infarction and related complications, such as major seizures and cerebral hernias, while post-stroke pneumonia and heart failure occur beyond the acute phase.

The pathophysiology of frailty is a decline in the physiological regulatory systems. This results in dynamic imbalance and impaired resilience, accompanied by an increased vulnerability to stressors. When a certain amount of dysregulation occurs, clinical manifestations of frailty occur with increased mortality and disability (31). When stress events occur (such as, in acute cerebral infarction), the functional ability of pre-frail or frail people deteriorates rapidly (17), increasing the risk of short-term mortality and acute disease severity (32).

Frailty after stroke is common, and the prevalence of post-stroke frailty is two times that of non-stroke patients (33). Approximately one out of four patients with acute stroke develop frailty, and four more patients develop frailty if their pre-stroke status is pre-frail (34). Our study found that pre-stroke frailty was not an independent influencing factor for 1-year mortality. A study with a mean follow-up time of 1.6 years shows older adults over 80 years had a significantly attenuated association between pre-stroke frailty and long-term survival after stroke, which may be associated with heterogeneity in older adults (27). Older adults usually combined with higher NIHSS and infection rates after acute cerebral infarction, which aggravates the deterioration of previous frailty status (33). Previous studies have shown that factors, such as advanced age, co-infection, and higher NIHSS score have a correlation with post-stroke long-term mortality (35–37), which may reduce the predictive effect of pre-stroke frailty on post-stroke long-term mortality. Therefore, paying attention to the prevention and treatment of infection in the acute phase can effectively reduce long-term mortality (38, 39).

Our findings emphasize that frailty was strongly associated with functional recovery, whether at 28 days or 1 year. Functional improvement was significantly reduced with pre-stroke frailty deterioration. This study did not exhibit post-stroke rehabilitation exercise as all patients underwent rehabilitation guidance and training during hospitalization at our center. There is a vicious cycle as well because pre-stroke frailty was significantly associated with stroke severity (26) and post-stroke neurologic impairment exacerbated deterioration to frailty (40). High frailty risk was independently associated with a decreased likelihood of favorable 3-month outcomes in patients with acute ischemic stroke who underwent endovascular stroke treatment (41). Simultaneously, in participants with mild stroke, health-related quality-of-life was impaired and continued to deteriorate among patients with in-hospital frailty from 3 to 18 months post-stroke (42).

Frailty is highly prevalent and is associated with adverse outcomes and increased healthcare costs. Pre-frailty is a dynamic process from quantitative to qualitative change between robust and frail individuals, who have adverse risk factors but are not yet undergoing severe physical and physiological changes associated with frailty (31). The transition time between pre-frailty to frailty in this study is uncertain. It may be that frailty and vascular changes are already associated before the onset of an overt cerebrovascular event (33) and the acute shock exacerbated further the patient's frailty status. Of note, it is clear that prolonged bouts of sedentary lifestyle and anorexia increase the incidence of frailty in the elderly (31, 43–45). Primary care interventions that promote physical activity and nutrition may arrest the progression of pre-frailty to frailty (46), reducing the likelihood of adverse outcomes after stroke.

This study cannot determine whether morbidity in cerebral infarction is greater in frail than non-frail patients, but existing studies have demonstrated that frailty is a high-risk factor for severe stroke and increased frailty severity (47, 48). Advocating for the elderly to increase physical activity, conduct moderate exercise, and increase intake of high-quality protein and trace elements is a powerful measure to prevent acute cerebrovascular events and reduce mortality and severe disability (49, 50).

There were several limitations in interpreting the results of this cohort study. First, this was a single-center study with a small sample size. Second, the applied frailty research tool was the FRAIL score. This puts the study at risk of notification error because subjects who can answer questions independently are prone to recall bias, while some patients who cannot express themselves provide relevant information through their guardians or caregivers, which is also prone to recall bias. Patients were likely to exaggerate the feelings of fatigue because of the illusion of temporary fatigue before the onset. It may increase the prevalence of fatigue and frailty before the stroke. Third, this study did not investigate the incidence of frailty after the stroke, and further research is needed to investigate the effect of pre-stroke frailty on post-stroke frailty.

## Conclusion

In conclusion, our study suggests that pre-stroke frailty status is directly associated with poor outcomes after acute cerebral infarction. It was an independent influencing factor for 28-day mortality but not for 1-year mortality and was associated with severe disability at 28 days and 1 year. More evidence-based studies are needed to develop interventions that may reverse pre-frailty.

## Data Availability Statement

The raw data supporting the conclusions of this article will be made available by the authors, without undue reservation.

## Ethics Statement

The studies involving human participants were reviewed and approved by the Ethics Committee of Huadong Hospital. The patients/participants provided their written informed consent to participate in this study. Written informed consent was obtained from the individual(s) for the publication of any potentially identifiable images or data included in this article.

## Author Contributions

FY, WW, and AY conceived the project and designed the study. FY, NL, LY, and WW contributed to participant recruitment, data collection, and data analysis. FY, NL, JC, and AY wrote the article. All authors contributed to the article and approved the submitted version.

## Funding

This work was supported by Shanghai Sailing Program (Grant Numbers: 21YF1411600) and Shanghai Municipal Key Clinical Specialty (Grant Numbers: shslczdzk02801).

## Conflict of Interest

The authors declare that the research was conducted in the absence of any commercial or financial relationships that could be construed as a potential conflict of interest.

## Publisher's Note

All claims expressed in this article are solely those of the authors and do not necessarily represent those of their affiliated organizations, or those of the publisher, the editors and the reviewers. Any product that may be evaluated in this article, or claim that may be made by its manufacturer, is not guaranteed or endorsed by the publisher.
