# Supplementary material for: Association of Pre-stroke Frailty With Prognosis of Elderly Patients With Acute Cerebral Infarction: A Cohort Study
Source: Front Neurol. 2022 May 30;13:855532. doi: 10.3389/fneur.2022.855532 (PMC9196308; doi:10.3389/fneur.2022.855532)
Supplement: Supplementary file 1 [file Data_Sheet_1.PDF]

Attached table1. Demographics and clinical characteristics of the participants for survival analysis

	Robust (n=97)	Pre-frail (n=108)	Frail (n=56)	P-value
Age[year,median(IQR)]	75.0(70-79)	75.5(69-80)	80.5(73-86)	**<0.001***
BMI[kg/m2,median(IQR)]	24.4(23.9-25.9)	24.4(22.5-25.3)	24.6(23.4-26.1)	0.161
NIHSS score[median(IQR))	2(1-7)	5(2-10)	7(2-12)	**0.014***
Sex				0.259
Male(%)	60(23.0)	62(23.7)	27(10.4)	
Female(%)	37(14.2)	46(17.6)	29(11.1)	
TOAST				**0.010***
Atherosclerotic(%)	20(7.7)	28(10.7)	15(5.7)	
Lacunar(%)	32(12.3)	40(15.3)	10(3.8)	
Cardioembolic(%)	19(7.3)	21(8.0)	18(7.0)	
Unknown(%)	26(10.0)	17(6.5)	9(3.4)	
Other(%)	0(0.0)	2(0.8)	4(1.5)	
Side of lesion				0.216
Left(%)	53(20.2)	43(16.5)	27(10.3)	
Right(%)	32(12.3)	45(17.1)	18(6.9)	
Both(%)	11(4.2)	19(7.3)	11(4.2)	
Stroke treatment				0.188
Antiplatelet therapy(%)	88(33.8)	97(37.2)	52(19.9)	
Intravenous thrombolysis(%)	9(3.4)	6(2.3)	3(1.1)	
Thrombectomy(%)	0(0.0)	5(1.9)	1(0.4)	

Previous stroke(%)	21(8.0)	37(14.2)	20(7.6)	0.081
Smoking				0.177
Former(%)	15(5.7)	18(6.9)	2(0.8)	
Current(%)	11(4.2)	14(5.4)	9(3.4)	
Never(%)	71(27.3)	76(29.1)	45(17.2)	
Hypertension(%)	78(29.9)	83(31.8)	50(19.2)	0.157
Diabetes Mellitus(%)	37(14.2)	50(19.2)	25(9.6)	0.479
Hyperlipidemia(%)	30(11.5)	24(9.2)	15(5.7)	0.369
Atrial fibrillation(%)	24(9.2)	21(8.0)	19(7.3)	0.123
Concurrent infection(%)	13(5.0)	30(11.5)	22(8.4)	**0.001**[*]

NIHSS,National Institutes of Health Stroke Scale.

Values are shown as median(interquartile range)or ordinal variables and counts(%)for categorical variables.

[*]Indicates a significant difference(p<0.05)between non-frail and frail.

Attached table2. Cox regression for 28-day and one year follow-up survival status for survival analysis

	28-day survival status		One-year survival status	
	HR(95%CI)	P-value	HR(95%CI)	P-value
Age	1.00(0.92-1.01)	0.727	1.07(1.03-1.12)	**0.002**[*]
Sex	1.48(0.49-4.48)	0.487	0.81(0.42-1.54)	0.515
Concurrent infection	0.32(0.07-1.44)	0.138	0.19(0.08-0.44)	**< 0.001**[*]
NIHSS	1.11(1.02-1.20)	**0.009**[*]	1.07(1.02-1.12)	**0.003**[*]

Frailty status				
Robust	Reference	-	Reference	-
pre-frail	0.58(0.14-2.50)	0.466	0.91(0.42-1.96)	0.801
Frail	1.83(0.51-6.65)	0.356	1.37(0.63-3.02)	0.430
Previous stroke	0.39(0.10-1.52)	0.175	0.91(0.45-1.84)	0.781
TOAST				
Atherosclerotic	Reference	-	Reference	-
Lacunar	0.00(0.00-3.96E+149)	0.954	0.23(0.03-1.89)	0.171
Cardioembolic	2.10(0.61-7.23)	0.238	1.12(0.55-2.27)	0.760
Unknown	1.08(0.09-12.89)	0.950	2.31(0.86-6.30)	0.097
Other	2.76(0.25-29.98)	0.405	1.03(0.22-4.81)	0.967

*Indicates a significant difference(p<0.05)between the groups.

Attached table 3.Survival status for 28-day and one year follow-up for survival analysis

		All (n=261)	Robust (n=97)	Pre-frail (n=108)	Frail (n=56)	X^2-test	
						X^2-value	p-value
28-day survival status	Survival	246	93(95.9%)	104(96.3%)	49(87.5%)	6.019	0.049*
	Death	15	4(4.1%)	4(3.7%)	7(12.5%)		
One-year survival status	Survival	215	85(87.6%)	91(84.3%)	39(69.6%)	8.361	0.015*
	Death	46	12(12.4%)	17(15.7%)	17(30.4%)		

NIHSS,National Institutes of Health Stroke Scale.

Values are shown as median(interquartile range)or ordinal variables and counts(%)for categorical variables.

*Indicates a significant difference($p<0.05$)between non-frail and frail.
